# Neuropsychological Profiles in Genetic Frontotemporal Dementia: A Meta-Analysis and Systematic Review

**DOI:** 10.14336/AD.2024.0183

**Published:** 2025-06-24

**Authors:** Jackie M. Poos, Esther van den Berg, Liset de Boer, Sabrina Meertens-Gunput, Elise G.P. Dopper, Harro Seelaar, Lize C. Jiskoot

**Affiliations:** ^1^Department of Neurology and Alzheimer Center Erasmus MC, Erasmus MC University Medical Center, Rotterdam, the Netherlands.; ^2^Department of Research and Education, Medical Library, Erasmus Medical Center, Rotterdam, the Netherlands.; ^3^Dementia Research Center, University College London, Queen Square, London WC1N 3AR, United Kingdom

**Keywords:** frontotemporal dementia, attention, executive function, memory, language, social cognition

## Abstract

Characterization of cognitive profiles across genetic FTD gene mutations is crucial for the identification of sensitive endpoints for clinical trials targeting specific pathologies. However, no systematic overview of the literature describing cognitive profiles in different FTD gene mutations has been made thus far. We performed a meta-analysis and systematic review to characterize cognitive profiles across the different FTD gene mutations and clinical disease stages of familial frontotemporal dementia (FTD). We included 27 studies comparing presymptomatic (n=1027), and/or symptomatic (n=574) mutation carriers (*GRN, MAPT, C9orf72*) with controls (n=1296). We extracted cognitive data and grouped them into six cognitive domains (language, attention and mental processing speed, executive function (EF), memory, social cognition, and visuospatial abilities). These domains were further subdivided into specific cognitive sub-processes. We calculated Hedges’ *g* and performed multilevel meta-analyses per cognitive domain and FTD gene mutation comparing presymptomatic and symptomatic mutation carriers to controls. Moderator analyses were performed to the effect of age, education, sex, and cognitive subprocess. Eleven studies into rarer FTD mutations were included in the systematic review. Presymptomatic *GRN* mutation carriers showed deficits in EF, and presymptomatic *C9orf72* mutation carriers in language, EF, and attention. Presymptomatic *MAPT* mutation carriers did not differ from controls on any of the cognitive domains. All symptomatic mutation carriers had deficits in language, EF, attention, and memory. Both in the presymptomatic and symptomatic stage cognitive sub-processes for language, attention and mental processing speed, EF, and memory were differentially affected in *GRN, MAPT*, and *C9orf72*. Cognitive decline was present in the presymptomatic stage of *GRN* and *C9orf72* mutation carriers, but not *MAPT* mutation carriers. Unique cognitive sub-processes were affected in *GRN, MAPT*, and *C9orf72*. This study increased our knowledge of the cognitive deficits in familial FTD, which can aid in differential diagnosis and selection of endpoints for clinical trials.

## 1. Background

**Figure 1.**
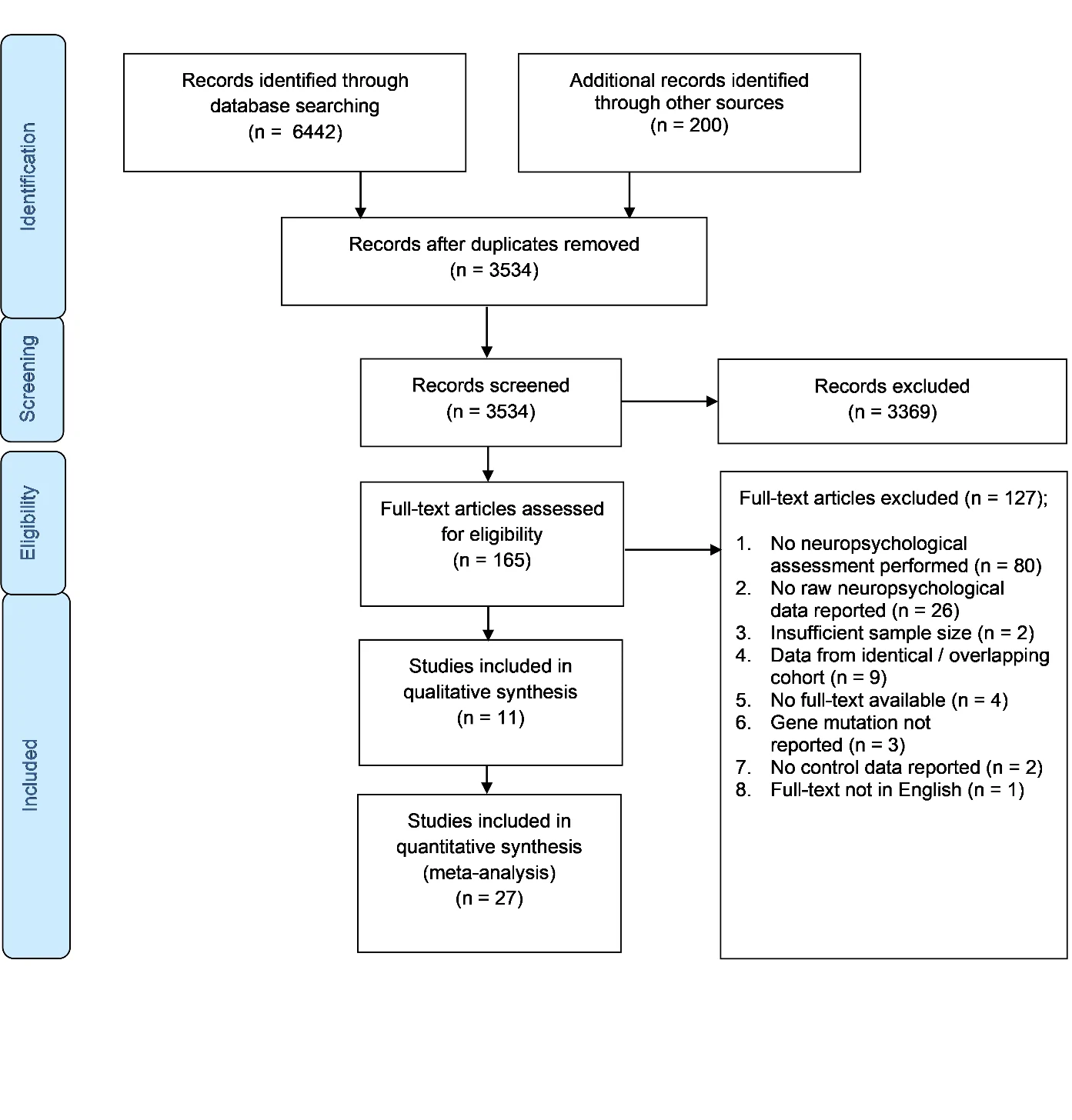
PRISMA flowchart of the literature search.

Frontotemporal lobar degeneration (FTLD) is a clinically and pathologically heterogeneous type of early-onset dementia, typically characterized by atrophy of the frontal and/or temporal lobes [1, 2]. Behavioral variant frontotemporal dementia (bvFTD) is the most common clinical presentation, although all clinical phenotypes within the FTLD spectrum can be observed, ranging from amyotrophic lateral sclerosis (ALS), language syndromes (primary progressive aphasia - PPA), to atypical parkinsonism (corticobasal syndrome, CBS - and progressive supranuclear palsy, PSP) [3-8] (Fig. 1). FTLD has a strong genetic component, with approximately 40-60% of cases having a positive family history for dementia, and up to 40% having an autosomal dominant pattern of inheritance [3, 4, 9]. Mutations in the progranulin (*GRN*) and microtubule-associated protein tau (*MAPT*) genes and repeat expansion in chromosome 9 open reading frame 72 (*C9orf72*) are the three most common causes of familial FTLD [4], each accounting for ~5-10% of cases [5]. Accounting for ~1-2%, mutations in TANK-binding kinase 1 (*TBK1*) are now being identified as the fourth most common cause [5], followed by the 43-kDA transactive response (TAR)-DNA-binding protein (*TARDBP*) mutation, which is thought to cause <2% of familial FTLD [10, 11]. Together with other rare mutations - valosin-containing protein, *VCP*), charged multivesicular body protein 2B (*CHMP2B*), and fused in sarcoma (*FUS*) - it cumulatively accounts for the last <5% of familial FTLD [5].

*GRN* mutations typically lead to prominent asymmetrical atrophy of the frontal, temporal, and parietal lobes, most frequently resulting in a clinical diagnosis of bvFTD or nfvPPA and may be accompanied by parkinsonism [5]. *MAPT* mutations are primarily associated with atrophy of the anterior temporal lobe [5]. bvFTD is the main phenotype, but may be accompanied by CBS or PSP [5]. The *C9orf72* repeat expansion often leads to a diffuse pattern of brain atrophy, affecting not only the frontal and temporal regions but also extending to posterior cortical and subcortical areas, including the cerebellum [5]. This usually leads to a clinical diagnosis of bvFTD and/or ALS [5]. In the rarer mutations, atrophy of frontotemporal regions is most apparent, with additional parietal involvement being described in particularly *TBK1, CHMP2B*, and *FUS* [12]. bvFTD is the most common phenotype, although cases of (FTD-)ALS, CBS, PSP, as well as nfvPPA or svPPA have also been reported [12].

Sometimes being overshadowed by marked decline in personality and behavior, cognitive disabilities are thought to be an early symptom of the symptomatic disease stage of FTLD. However, an increasing number of studies are describing the emergence of subtle cognitive deficits as early as the presymptomatic stage, starting around 10 years prior, with more rapid decline from the symptomatic onset of disease onwards [13-15]. For example, early decline in memory, language, and social cognition has been described in *MAPT* mutation carriers, whereas early decline in attention [14-18], executive function, and social cognition has been found in *GRN* and *C9orf72* mutation carriers [15, 19-22]. Yet, other studies have failed to find these results [23, 24]. For the rarer mutations, the majority of studies into neuropsychological deficits have included case or small family-based series. No study has comprehensively characterized and compared cognitive profiles between autosomal dominant mutations across the entire FTLD spectrum. Quantifying the nature and extent of cognitive deficits in familial FTLD can increase our understanding and recognition of the specific cognitive deficits accompanying these mutations. This can aid earlier and better differential diagnosis and improve the selection of the most sensitive clinical endpoints for upcoming disease-modifying trials. In this systematic and meta-analytic study, we examined psychometric performance in the six major cognitive domains (language, attention and mental processing speed, executive function, memory, social cognition, and visuospatial abilities) across mutations (*GRN, MAPT, C9orf72, TARDBP, TBK1, VCP, CHMP2B* and *FUS*) and clinical (presymptomatic and symptomatic) disease stages of familial FTLD. Considering the neuroimaging profile and clinical phenotypes associated with each genetic mutation (5, 12], we hypothesize early deficits in memory and semantics in *MAPT* mutation carriers, and attention and executive function deficits in *GRN* and *C9orf72* mutation carriers. It is expected that social cognition is impaired in all genetic variants.

## 2. Methods

### 2.1 Study identification and selection

In collaboration with the Erasmus MC University Medical Center Medical Library, the appropriate MeSH and entry terms for this study were identified. An exhaustive search strategy was developed by an experienced information specialist (SMG) in cooperation with the lead author (LCJ). Consequently, we performed a MedLine, Embase and PsycINFO literature search covering the period January 1806 to July 2023 (see Appendix A for an overview of the search terms used and Figure 1 for the PRISMA flowchart of the literature search). The search contained terms for 1) frontotemporal dementia, 2) genetics/heredity and 3) cognition and (neuro) psychological aspects. Reviews, editorials, letters, conference abstracts, books (including dissertations) or book chapters, and animal studies were excluded from the search. A total of 3534 articles were identified in this search. The articles were imported into EndNote and duplicates were removed by the experienced information specialist (SMG) using the method as described by Bramer et al. [25]. The titles and abstracts of these articles were assessed for relevance by two independent raters (LCJ and JMP), and potentially eligible articles were retrieved in full-text. The reference lists of the identified articles were also examined for additional relevant articles. The methods in this review are described based on the "Preferred reporting items for systematic reviews and meta-analyses (Prisma) Checklist" [26] and the Prisma-S extension to the PRISMA Statement for Reporting Literature Searches in Systematic Reviews [27].

Articles that met the following criteria were included in our study:

1. The study was an original English language article;
2. Cognitive performance was assessed using at least one validated neuropsychological test in one of the six specified major domains (language, attention and mental processing speed, executive function, memory, social cognition, and/or visuospatial abilities) in at least one FTLD mutation (*GRN, MAPT, C9orf72, TARDBP, TBK1, VCP, CHMP2B*, and/or *FUS*) compared to a healthy (i.e., cognitively unimpaired) control group;
3. Sufficient information regarding psychometric test performance (i.e., means, standard deviations (SDs), effect sizes) were provided to perform a meta-analysis;
4. Sample sizes were n≥5 in studies describing *GRN, MAPT*, and *C9orf72* mutation carriers. Due to the rarity of the other mutations, case studies and smaller case series were also included for the *TBK1, TARDBP, VCP, CHMP2B*, and *FUS* genes.

Studies were only included in the meta-analysis or systematic review when the two independent raters both agreed on eligibility. Disagreements were resolved through discussion until consensus about inclusion or exclusion was reached. If there was a possibility that multiple studies were describing a similar cohort of subjects, the study’s principal investigator was contacted to comment on possible overlap. When other test scores than raw means and SDs were reported (such as z-scores or percentiles), the study’s principal investigator was also approached to send the raw neuropsychological test scores. Allowing a maximum of 10% overlap between cohorts [28], we only included the study with the largest sample size and/or set of outcome variables. From longitudinal studies, only baseline neuropsychological data were used. Our study was performed in accordance with the declaration of Helsinki for medical research involving human subjects and followed the PRISMA guidelines for systematic reviews and meta-analyses [26]. No additional medical ethical approval was needed as we only made use of previously published data.

### 2.2 Data extraction

After the final study selection, we extracted the following demographic and clinical patient data from the articles: age, sex, years of formal education, clinical status and diagnosis, gene mutation, disease duration, global cognitive performance by means of the Mini-Mental State Examination (MMSE) [29], and diagnostic criteria used (i.e., Neary et al., 1998 [1], McKhann et al., 2001 [2], Rascovsky et al., 2011 [3], or Miller et al., 1997 [30] for bvFTD, Strong et al., 2009 [9], or Brooks et al., 2000 [31] for ALS, Gorno-Tempini et al., 2011 [32] for PPA, and Armstrong et al., 2014 [33] for CBD), if available. We then grouped the neuropsychological tests used in the selected articles into six cognitive domains according to standard neuropsychological practice: language, attention and mental processing speed, executive function, memory, social cognition, and visuospatial abilities (Appendix B). These six domains were further subdivided into specific cognitive sub-processes, e.g., the language domain was categorized into fluency, naming, and semantic processing (Appendix B). Neuropsychological test scores (means, SDs), and number of participants per patient and control group(s), were extracted after.

### 2.3 Data analysis

We performed statistical analyses using the *metafor* package in R version 4.0.4 (RStudio, PBC, Boston, MA). To deal with interdependency of effect sizes due to studies reporting multiple independent outcomes, we used multilevel meta-analysis including random factors for study and effect size (i.e. cognitive outcome). This three-level structure model allows effect sizes to vary between participants (Level 1), outcomes extracted from the same study (Level 2), and between studies (Level 3) [34]. We examined differences in cognitive domains for studies reporting on (1) presymptomatic mutation carriers and controls, and (2) symptomatic mutation carriers and controls. Hedges’ *g* was calculated, expressing the magnitude of the difference between mutation carriers and controls. A negative effect size indicates worse performance in mutation carriers compared to controls. To determine whether within-study variance (Level 2) and between-study variance (Level 3) was significant, two separate one-sided log-likelihood-ratio tests were performed. In this test, the fit of the original three-level model is compared to the fit of a two-level model in which either within-study or between-study variance is no longer modeled. Significant within-study or between-study variance implies that there is more variability in effect sizes (within and between studies) than may be expected from sampling variance (Level 1) alone. In that case, moderator analyses were performed to test study (mean age, mean education level, mean percentage females) and effect size (cognitive sub-process; Appendix B) characteristics by extending the three-level random effects model to a mixed effects model. Egger’s regression analysis was performed, and funnel plot asymmetry was evaluated as indications for publication bias.

## 3. Results

Of the 3534 articles identified in the literature search, 3369 were excluded after reviewing the titles and abstracts for eligibility. Full text versions were retrieved for the remaining 165 articles, of which 38 were eligible for study inclusion: 27 studies could be used in the quantitative synthesis (meta-analysis), whereas 11 studies were used in the qualitative synthesis (systematic review) (Fig. 1). Table 1 lists the characteristics of the studies included in the quantitative synthesis.

### 3.1 Meta-analysis

Study characteristics, demographic and clinical data are reported in Tables 1-2.

#### 3.1.2 Cognitive profile of GRN mutation carriers

##### 3.1.2.1 The presymptomatic stage

**Figure 2.**
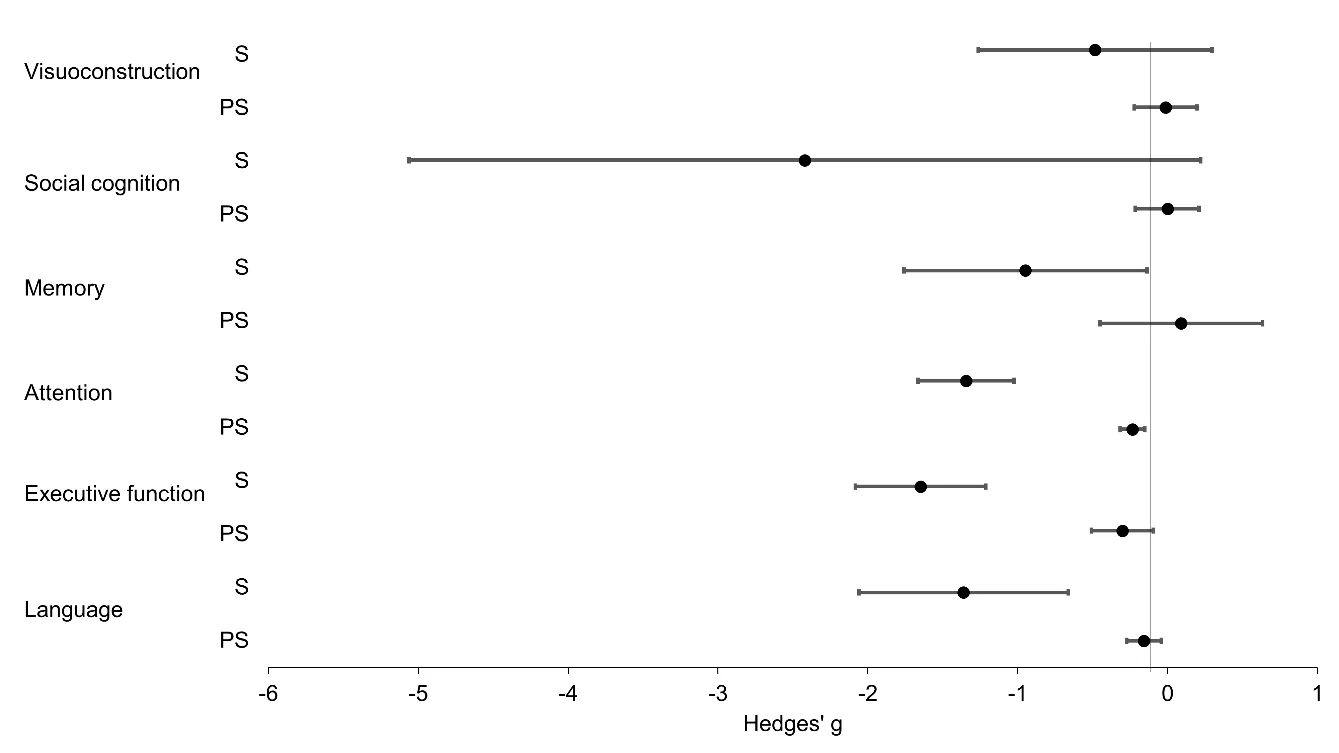
Forest plot illustrating overall effect sizes and bias-corrected 95% confidence intervals for each cognitive domain comparing presymptomatic and symptomatic *GRN* mutation carriers to controls.

A significant difference between presymptomatic mutation carriers and controls was found for executive function, but no other cognitive domains (Fig. 2). Executive function was assessed in nine studies comprising 31 effect sizes and a small effect size of -0.20 (95% CI [-0.35, 0.05]; *t*(30) = -2.68, *p*=0.010) was found. There was no evidence for within-study or between-study variance (*p*>0.05).

**Table 1:** Characteristics of studies included in the meta-analysis.

Study	Carriers	Controls	Age	Sex	Education	Diagnosis	Criteria	Disease duration	MMSE	Neuropsychological tests used
Symptomatic *GRN* mutation carriers										
Olney et al. (2020) (67)	43	102	61.9 (9.0)	58.1	14.5 (2.8)	bvFTD, PPA, CBS, other	NR	NR	NR	Craft immediate and delayed recall, CVLT, Benson Figure, MINT, semantic and letter fluency, Digit Span forwards and backwards, TMT A and B
Poos et al. (2020) (51)	20	24	60.4 (7.4)	60.0	5.0 (2.0)*	bvFTD	NR	1.0 (1.1)	22.5 (6.3)	TMT A and B, Stroop CWT, mWCST, BNT, semantic and letter fluency, RAVLT, RBMT, VAT, Digit Span forwards and backwards WAIS-III, clock drawing
Poos et al. (2021) (17)	27	290	60.8 (7.9)	37.0	12.0 (3.5)	bvFTD, PPA, ALS	3, 31, 32	NR	22.9 (6.8)	FCSRT
Poos et al. (2022) (68)	46	255	63.6 (7.9)	50.0	11.9 (3.3)	bvFTD, PPA, ALS	3, 31, 32	NR	20.2 (7.6)	WMS-R digit span, TMT A and B, WAIS symbol digit, D-KEFS Stroop CWT, modified Camel and Cactus Test, BNT30, verbal fluency, Benson Figure, Facial Emotion Recognition
Moore et al. (2020) (18)	33	248	63.9 (8.4)	48.0	11.3 (3.3)	bvFTD	NR	NR	NR	Modified Camel and Cactus Test
Russell et al. (2022) (22)	32	246	64.2 (8.4)	47.0	11.6 (3.6)	bvFTD, PPA, other	NR	NR	21.8 (6.3)	Facial Emotion Recognition, Faux Pas
Symptomatic *MAPT* mutation carriers										
Deters et al. (2014) (69)	8	8	50.1 (5.4)	37.5	12.75 (0.9)	bvFTD	1-2	NR	23.6 (5.6)	Word fluency, Block Design, CERAD Word List Immediate and Delayed recall
Moore et al. (2020) (18)	15	248	59.7 (6.0)	47.0	14.9 (3.8)	bvFTD, PPA, dementia NOS	NR	NR	NR	Modified Camel and Cactus Test
Olney et al. (2020) (67)	45	102	53.3 (10.0)	51.1	15.3 (2.3)	bvFTD, PPA, CBS, other	NR	NR	NR	Craft immediate and delayed recall, CVLT, Benson Figure, MINT, semantic and letter fluency, Digit Span forwards and backwards, TMT A and B
Poos et al. (2020) (51)	31	24	62.1 (9.1)	41.9	5.0 (2.0)*	bvFTD	NR	3.1 (2.7)	26.5 (2.7)	TMT A and B, Stroop CWT, mWCST, BNT, semantic and letter fluency, RAVLT, RBMT, VAT, Digit Span forwards and backwards WAIS-III, clock drawing
Poos et al. (2021) (17)	17	290	58.6 (6.8)	41.2	14.5 (3.9)	bvFTD, PPA, ALS	3, 31, 32	NR	26.2 (3.1)	FCSRT
Poos et al. (2022) (68)	24	255	57.3 (10.2)	33.3	13.7 (3.9)	bvFTD, PPA, ALS	3, 31, 32	NR	23.7 (6.7)	WMS-R digit span, TMT A and B, WAIS symbol digit, D-KEFS Stroop CWT, modified Camel and Cactus Test, BNT30, verbal fluency, Benson Figure, Facial Emotion Recognition
Russell et al. (2022) (22)	18	246	59.8 (6.0)	56.0	14.6 (3.6)	bvFTD, other	NR	NR	23.2 (6.5)	Facial Emotion Recognition, Faux Pas
Spina et al. (2008) (70)	7	3	48.5 (4.4)	57.1	NR	bvFTD	1-2	2.1 (1.0)	25.6 (2.3)	Semantic and letter fluency, Digit Span forwards and backwards, Word List memory & recall, Logical Memory, Visual Reproduction & Recall, BNT, Block Design, constructional praxis, TMT A and B, WCST, Stroop CWT
Symptomatic *C9orf72* repeat expansion carriers										
Haapanen et al. (2020) (71)	5	13	67.2 (11.6)	60.0	12.0 (4.0)	PPA	1	2.2 (1.7)	16.0 (11.0)	CERAD-NB: semantic fluency, abbreviated BNT, word list learning, recall and recognition, constructional praxis, clock drawing
Irish et al. (2013) (72)	8	15	60.4 (4.6)	25.0	10.4 (1.8)	bvFTD, FTD-MND	3-4	5.1 (3.2)	NR	TMT A and B, letter fluency, WMS3 digit span forwards and backwards, Hayling test
Jiskoot et al. (2020) (21)	6	49	59.8 (9.0)	50.0	5.0 (0.6)*	bvFTD	3	5.4 (4.0)	27.5 (3.8)	ERT
Lee et al. (2014) (73)	14	14	58.3 (7.7)	28.6	14.9 (3.7)	bvFTD, ALS	1, 3, 78	7.8 (7.9)	25.2 (3.5)	CVLT, Benson Figure, Digit Span backwards, TMT A and B, design fluency, semantic and letter fluency, abbreviated BNT
Ly et al. (2019) (74)	9	18	63.0 (4.0)	44.0	NR	ALS	NR	2.5 (1.1)	27.4 (3.0)	TMT A and B, Letter Number Sequencing, semantic fluency, logical memory WMS-III
Moore et al. (2020) (18)	56	248	62.2 (7.8)	34	13.0 (3.9)	bvFTD, PPA, FTD-ALS, PSP, dementia NOS	NR	NR	NR	Modified Camel and Cactus Test
Olney et al. (2020) (67)	99	102	58.6 (11.2)	54.5	15.3 (2.5)	bvFTD, PPA, CBS, other	NR	NR	NR	Craft immediate and delayed recall, CVLT, Benson Figure, MINT, semantic and letter fluency, Digit Span forwards and backwards, TMT A and B
Poos et al. (2020) (51)	29	24	52.6 (5.5)	34.5	5.0 (2.0)*	bvFTD	NR	1.4 (2.0)	25.9 (2.9)	TMT A and B, Stroop CWT, mWCST, BNT, semantic and letter fluency, RAVLT, RBMT, VAT, Digit Span forwards and backwards WAIS-III, clock drawing
Poos et al. (2021) (17)	52	290	62.0 (7.6)	36.5	12.8 (3.3)	bvFTD, PPA, ALS	3, 31, 32	NR	25.3 (3.9)	FCSRT
Poos et al. (2022) (68)	66	255	62.2 (8.9)	36.4	13.2 (3.7)	bvFTD, PPA, ALS	3, 31, 32	NR	23.7 (6.1)	WMS-R digit span, TMT A and B, WAIS symbol digit, D-KEFS Stroop CWT, modified Camel and Cactus Test, BNT30, verbal fluency, Benson Figure, Facial Emotion Recognition
Russell et al. (2022) (22)	53	246	62.3 (8.0)	64.0	13.0 (3.6)	bvFTD, PPA, FTD-MND, PSP, other	NR	NR	24.7 (4.9)	Facial Emotion Recognition, Faux Pas
Saxon et al. (2020) (75)	13	30	NR	NR	NR	bvFTD | FTD-ALS	3, 32	NR	NR	Graded Naming test, Pyramids and Palm trees, semantic and letter fluency, D-KEFS sorting test, Brixton test, Hayling test, Ekman faces
Presymptomatic *GRN* mutation carriers										
Barandiaran et al. (2012) (19)	13	19	49.9 (12.8)	53.8	15.4 (3.3)	N/A	N/A	N/A	28.6 (1.5)	WAIS-III Block Design, TMT A and B, WCST, semantic and letter fluency, abbreviated BNT, CERAD verbal learning test
Borroni et al. (2008) (11)	7	15	37.0 (12.0)	42.8	8.5 (2.2)	N/A		N/A	30.0 (0.0)	Digit Span, semantic and letter fluency, TMT A and B
Hallam et al. (2014) (20)	8	16	47.3 (9.8)	87.5	13.0 (2.3)	N/A	N/A	N/A	29.8 (2.3)	TMT A and B, Stroop CWT, Digit Span forwards and backwards, spatial span forwards and backwards, digit symbol, BNT, semantic and letter fluency, RCF, Block Design, Visual Reproduction, CVLT, logical memory, Design Fluency, WCST
Jiskoot et al. (2016) (14)	30	39	52.9 (8.5)	63.3	5.7 (0.9)*	N/A	N/A	N/A	29.0 (1.6)	BNT, SAT verbal, semantic and letter fluency, TMT A and B, Stroop CWT, LDST, WCST, WAIS Digit Span and Block Design, Ekman Faces, Happé cartoons, RAVLT, VAT, clock drawing
Jiskoot et al. (2020) (21)	22	49	53.3 (11.7)	63.6	5.6 (0.7)*	N/A	N/A	N/A	29.5 (0.8)	ERT
Lee et al. (2019) (76)	17	30	53.6 (11.5)	58.8	16.8 (3.6)	N/A	N/A	N/A	28.5 (1.1)	CVLT, Benson Figure, abbreviated BNT, Digit Span forwards and backwards, TMT A and B, Stroop CWT, semantic, letter and design fluency
Moore et al. (2020) (18)‡	79/53	248	39.2 (8.2) / 58.4 (7.8)	68.0 / 51.0	15.0 (3.7) / 14.2 (3.4)	N/A	N/A	N/A	NR	Modified Camel and Cactus Test
Olney et al. (2020) (67)	37	102	50.0 (14.7)	40.5	15.8 (2.8)	N/A	N/A	N/A	NR	Craft immediate and delayed recall, CVLT, Benson Figure, MINT, semantic and letter fluency, Digit Span forwards and backwards, TMT A and B
Pievani et al. (2014) (77)	5	5	45.0 (10.0)	60.0	11.0 (3.0)	N/A	N/A	N/A	29.0 (2.0)	RCF, BNT, semantic and letter fluency, RAVLT, TMT A and B
Poos et al. (2021) (17)	136	290	46.1 (12.4)	61.8	14.7 (3.5)	N/A	N/A	N/A	28.7 (4.6)	FCSRT
Poos et al. (2022) (68)	160	255	48.9 (12.7)	62.5	14.4 (3.7)	N/A	N/A	N/A	28.4 (4.9)	WMS-R digit span, TMT A and B, WAIS symbol digit, D-KEFS Stroop CWT, modified Camel and Cactus Test, BNT30, verbal fluency, Benson Figure, Facial Emotion Recognition
Russell et al. (2022) (22)	123	246	46.0 (11.8)	61.5	14.8 (3.6)	N/A	N/A	N/A	29.5 (0.9)	Facial Emotion Recognition, Faux Pas
Presymptomatic *MAPT* mutation carriers										
Cheran et al. (2019) (16)	12	32	48.8 (13.6)	66.7	15.3 (2.1)	N/A	N/A	N/A	28.5 (1.9)	Digit Span, WAIS Digit Symbol, TMT A and B, Design Fluency test, BNT, semantic and letter fluency, Semantic Associates Test, SRT, Benson Figure, SNQ
Geschwind et al. (2001) (78)	10	6	31.0 (8.0)	50.0	11.6 (-)	N/A	N/A	N/A	NR	TMT A and B, semantic fluency, WCST, Stroop CWT, Digit Span, Digit Symbol, RCF, BNT, RAVLT
Jiskoot et al. (2016)(14)	13	39	42.8 (10.8)	38.5	5.4 (1.2)*	N/A	N/A	N/A	29.5 (0.5)	BNT, SAT verbal, semantic and letter fluency, TMT A and B, Stroop CWT, LDST, WCST, WAIS Digit Span and Block Design, Ekman Faces, Happé cartoons, RAVLT, VAT, clock drawing
Jiskoot et al. (2020) (21)	7	49	48.6 (12.5)	71.4	4.7 (2.0)*	N/A	N/A	N/A	29.4 (0.8)	ERT
Moore et al. (2020) (18)	33/19	248	34.7 (7.0) / 50.0 (9.3)	64.0 / 58.0	14.7 (2.6) / 14.0 (3.7)‡	N/A	N/A	N/A	NR	Modified Camel and Cactus Test
Olney et al. (2020) (67)	48	102	37.3 (11.4)	56.3	15.8 (2.6)	N/A	N/A	N/A	NR	Craft immediate and delayed recall, CVLT, Benson Figure, MINT, semantic and letter fluency, Digit Span forwards and backwards, TMT A and B
Poos et al. (2021) (17)	56	290	39.8 (10.5)	60.7	14.5 (3.0)	N/A	N/A	N/A	29.5 (0.9)	FCSRT
Poos et al. (2022) (68)	62	255	42.5 (11.6)	62.9	14.0 (2.9)	N/A	N/A	N/A	29.5 (0.8)	WMS-R digit span, TMT A and B, WAIS symbol digit, D-KEFS Stroop CWT, modified Camel and Cactus Test, BNT30, verbal fluency, Benson Figure, Facial Emotion Recognition
Russell et al. (2022) (22)	49	246	39.8 (10.7)	63.3	14.6 (2.9)	N/A	N/A	N/A	29.6 (0.8)	Facial Emotion Recognition, Faux Pas
Presymptomatic *C9orf72* repeat expansion carriers										
Bertrand et al. (2018) (24)	41	39	39.8 (11.1)	58.5	NR	N/A	N/A	N/A	28.6 (1.3)	mini-SEA, Benson Figure, FCSRT, BNT, semantic and letter fluency
Jiskoot et al. (2020) (21)	18	49	43.0 (12.1)	55.6	5.6 (0.8)*	N/A	N/A	N/A	29.3 (0.8)	ERT
Lee et al. (2017) (79)	15	46	43.7 (10.2)	60.0	16.1 (1.5)	N/A	N/A	N/A	29.0 (1.2)	CVLT, Benson Figure, abbreviated BNT, Digit Span forwards and backwards, TMT A and B, Stroop CWT, semantic, letter and design fluency
Lulé et al. (2020) (55)	22	91	45.1 (11.9)	72.7	14.6 (3.1)	N/A	N/A	N/A	NR	Letter and design fluency, Doors B, TMT B
Montembeault et al. (2020) (80)	38	22	38.2 (8.0)	57.9	8/30†	N/A	N/A	N/A	28.7 (1.4)	Hayling test
Moore et al. (2020) (18)	68/40	248	39.9 (9.7) / 53.7 (8.9)	57.0 / 67.0	14.4 (2.4) / 14.4 (3.7)‡	N/A	N/A	N/A	NR	Modified Camel and Cactus Test
Olney et al. (2020) (67)	76	102	41.5 (12.8)	60.5	15.8 (2.1)	N/A	N/A	N/A	NR	Craft immediate and delayed recall, CVLT, Benson Figure, MINT, semantic and letter fluency, Digit Span forwards and backwards, TMT A and B
Papma et al. (2017) (23)	18	15	45.8 (13.8)	83.3	5.6 (0.8)*	N/A	N/A	N/A	30.0 (1.0)	BNT, SAT verbal, semantic and letter fluency, TMT A and B, Stroop CWT, LDST, Digit Span and Block Design, Ekman Faces, Happé cartoons, RAVLT, clock drawing
Poos et al. (2021) (17)	129	290	44.6 (11.1)	59.7	14.4 (3.0)	N/A	N/A	N/A	29.0 (2.1)	FCSRT
Poos et al. (2022) (68)	141	255	46.1 (11.2)	59.6	14.3 (2.8)	bvFTD, PPA, ALS	N/A	N/A	28.9 (2.6)	WMS-R digit span, TMT A and B, WAIS symbol digit, D-KEFS Stroop CWT, modified Camel and Cactus Test, BNT30, verbal fluency, Benson Figure, Facial Emotion Recognition
Russell et al. (2022) (22)	106	246	45.1 (11.5)	60.4	14.4 (2.9)	N/A	N/A	N/A	29.2 (1.1)	Facial Emotion Recognition, Faux Pas

Values indicate: sex: % females; disease duration (years); education (years); age and MMSE (SD) or [range]. Abbreviations: *C9orf72*, chromosome open reading frame 72; *GRN*, progranulin; *MAPT*, microtubule-associated protein tau; bvFTD, behavioral variant frontotemporal dementia; NOS, not otherwise specified; FTD-MND, frontotemporal dementia with motor neuron disease; PPA, primary progressive aphasia; PSP, progressive supranuclear palsy; CBS, corticobasal syndrome; MMSE, Mini-Mental State Examination; NR, not reported; N/A, not applicable; TMT, Trail Making Test; CWT, color word test; (m)WCST, (modified) Wisconsin Card Sorting Test; WAIS-III, Wechsler Adult Intelligence Scale Third Edition; BNT, Boston Naming Test; RAVLT, Rey Auditory Verbal Learning Test; RBMT; Rivermead Behavioural Memory Test; VAT, Visual Association Test; CERAD, Consortium to Establish a Registry for Alzheimer’s Disease; FCSRT, Free and Cued Selective Reminding Test; WMS3, Wechsler Memory Scale third edition; RCF, Rey-Osterrieth Complex Figure; CVLT, California Verbal Learning Test; D-KEFS, Delis-Kaplan Executive Function System; SRT, Selective Reminding Test; SNQ, Social Norms Questionnaire; mini-SEA, mini Social Cognition & Emotional Assessment; MINT, Multilingual Naming Test; ERT, Emotion Recognition Test; AAT, Aachener Aphasia Test.

* Level of education according to Verhage (81) (1= less than primary school, 7 = university degree).

† Reported as secondary / higher education.

‡ Reported as early/late presymptomatic. Diagnostic criteria: Neary et al. (1), McKhann et al. (2), Rascovsky et al. (3), Strong et al. (9), Brooks et al. (31), Gorno-Tempini et al. (32), Armstong et al. (33), McKhann et al. (82), Miller et al. (83).

##### 3.1.2.2. The symptomatic stage

Significant differences compared to controls were found in the domain’s language, executive function, attention and mental processing speed, and memory (Fig. 2). Language was assessed in four studies, comprising 12 effect sizes, and revealed a large effect size of -1.79 (95% CI [-2.24, -1.35]; *t*(11) = -8.91, *p*<0.001). There was evidence for significant between-study variance (*p*=0.006), but this was not moderated by the mean age, mean education level, sex distribution or cognitive sub-processes of the included primary studies.

Executive function was assessed in three studies, comprising nine effect sizes, and a large effect size of -2.10 (95% CI [-2.85, -1.35]; t(8) = -6.43, p<0.001) was found. Within-study variance was significant (p<0.001), and moderator analyses revealed that effect sizes were larger for cognitive flexibility tests than working memory tests (t(6) = -3.01, p=0.024). Attention and mental processing speed was assessed in three studies, comprising of 10 effect sizes, and a large effect size of -1.25 (95 % CI [-1.59, -0.91]; t(9) = -8.43, p<0.001) was found. There was evidence for significant within-study variance, and moderator analysis revealed that effect sizes were larger for information processing tests than attention tests (t(8) = -5.68, p<0.001). Memory was assessed in four studies, comprising 13 effect sizes, which revealed a large effect size of -1.65 (95% CI [-2.30, -1.01]; t(12) = -5.6, p<0.001). Both within-study and between-study variance were significant (p<0.05), which was moderated by the mean education level (t(38) = 9.70, p<0.001) of the included primary studies. No significant differences were found for social cognition or visuospatial functioning.

**Table 2:** Summary of demographic and clinical data of included studies in meta-analysis.

	*GRN*		*MAPT*		*C9orf72*	
	Presymptomatic	Symptomatic	Presymptomatic	Symptomatic	Presymptomatic	Symptomatic
***k***	12	6	9	8	11	12
***n***	690	201	309	165	712	410
**Sex**	59,62	50,02	59,18	45,64	62,68	42,5
**bvFTD, *n*** *	N / A	96/158	N / A	109/113	N / A	236/297
**PPA, *n*** *	N / A	57/158	N / A	0/113	N / A	10/297
**FTD-MND, *n*** *	N / A	0/158	N / A	0/113	N / A	39/297
**MND, *n*** *	N / A	0/158	N / A	0/113	N / A	9/297
**PSP** *	N / A	0/158	N / A	0/113	N / A	2/297
**NOS / other, *n*** *	N / A	2/158	N / A	4/113	N / A	11/297
**Age**	48,28 (11,07)	62,47 (8,17)	41,53 (10,54)	56,18 (7,24)	43,88 (11,08)	60,78 (7,81)
**Education level** *	13,96 (3,19)	12,26 (3,30)	14,31 (2,83)	14,29 (3,07)	14,80 (2,69)	13,08 (3,31)
**MMSE** *	29,10 (1,97)	21,85 (6,75)	29,10 (1,97)	24,80 (4,48)	29,09 (1,44)	24,46 (4,76)

Values indicate: k, number of included studies, n, number of individuals, sex: % females; age and MMSE, mean (SD), education (years). Abbreviations: *C9orf72*, chromosome open reading frame 72; *GRN*, progranulin; *MAPT*, microtubule-associated protein tau; bvFTD, behavioral variant frontotemporal dementia; PPA, primary progressive aphasia; FTD-MND, frontotemporal dementia with motor neuron disease; MND, motor neuron disease; PSP, progressive supranuclear palsy; NOS, not otherwise specified; MMSE, Mini-Mental State Examination; N/A, not applicable.

* Calculations based on the included studies that reported this data (see Table 1).

#### 3.1.3 Cognitive profile of MAPT mutation carriers

##### 3.1.3.1 The presymptomatic stage

**Figure 3.**
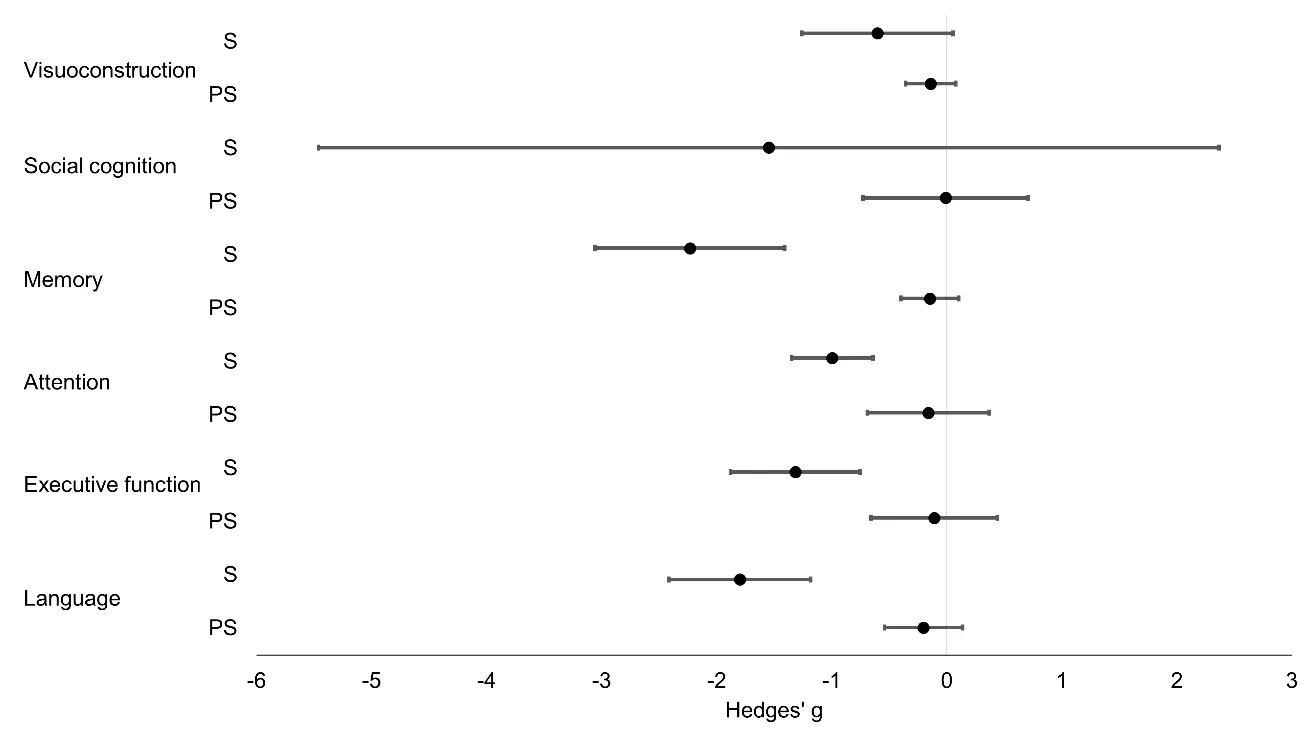
Forest plot illustrating overall effect sizes and bias-corrected 95% confidence intervals for each cognitive domain comparing presymptomatic and symptomatic *MAPT* mutation carriers to controls.

No significant differences were found between presymptomatic mutation carriers and controls (Fig. 3).

##### 3.1.3.2 The symptomatic stage

Significant differences compared to controls were found in the domains language, executive function, attention and mental processing speed, and memory (Fig. 3). Language was assessed in six studies, comprising 16 effect sizes, and a large effect size of -1.80 (95% CI [-2.42, -1.18]; *t*(15) = -6.23, *p*<0.001) was found. Within-study variance was significant (*p*<0.001), and moderator analysis revealed that effect sizes were larger for semantic processing tests than verbal fluency tests (*t*(13) = -2.13, *p*=0.038). In addition, there was trend evidence that effect sizes were larger for naming tests compared to verbal fluency tests (*t*(13) = -1.96, *p*=0.072). Executive function was assessed in four studies, comprising 13 effect sizes, and a large effect size of -1.32 (95 % CI [-1.88, -0.75]; *t*(12) = -5.09, *p*<0.001) was revealed. Within-study variance was significant (*p*<0.001), and moderator analysis revealed that effect sizes were larger for working memory tests than cognitive flexibility (*t*(10) = -2.87, *p*=0.017) and inhibitory control (*t*(10) = -3.26, *p*=0.009) tests. Attention and mental processing speed were also assessed in four studies, comprising 13 effect sizes, which revealed a large effect size of -1.00 (95% CI [-1.35, -0.64]; t(12) = -6.13, p<0.001). There was evidence for within-study variance (p<0.001), but this was not moderated by mean age, mean education level, sex distribution or cognitive sub-processes of the included primary studies. Memory was assessed in six studies, comprising 21 effect sizes, and a large effect size of -2.23 (95% CI [-3.06, -1.41]; t(20) = -5.65, p<0.001) was found. There was again evidence for within-study and between-study variance (p<0.007), but this was similarly not moderated by any of the study or effect size characteristics. Visuospatial abilities were assessed in five studies, comprising seven effect sizes, and there was trend evidence for impairment in mutation carriers compared to controls, with a moderate effect of -0.60 (95% CI [-1.26, 0.05]; *t*(6) = -2.25, *p*=0.066). No significant difference was found for social cognition.

#### 3.1.4 Cognitive profile of C9orf72 repeat expansion carriers

##### 3.1.4.1 The presymptomatic stage

**Figure 4.**
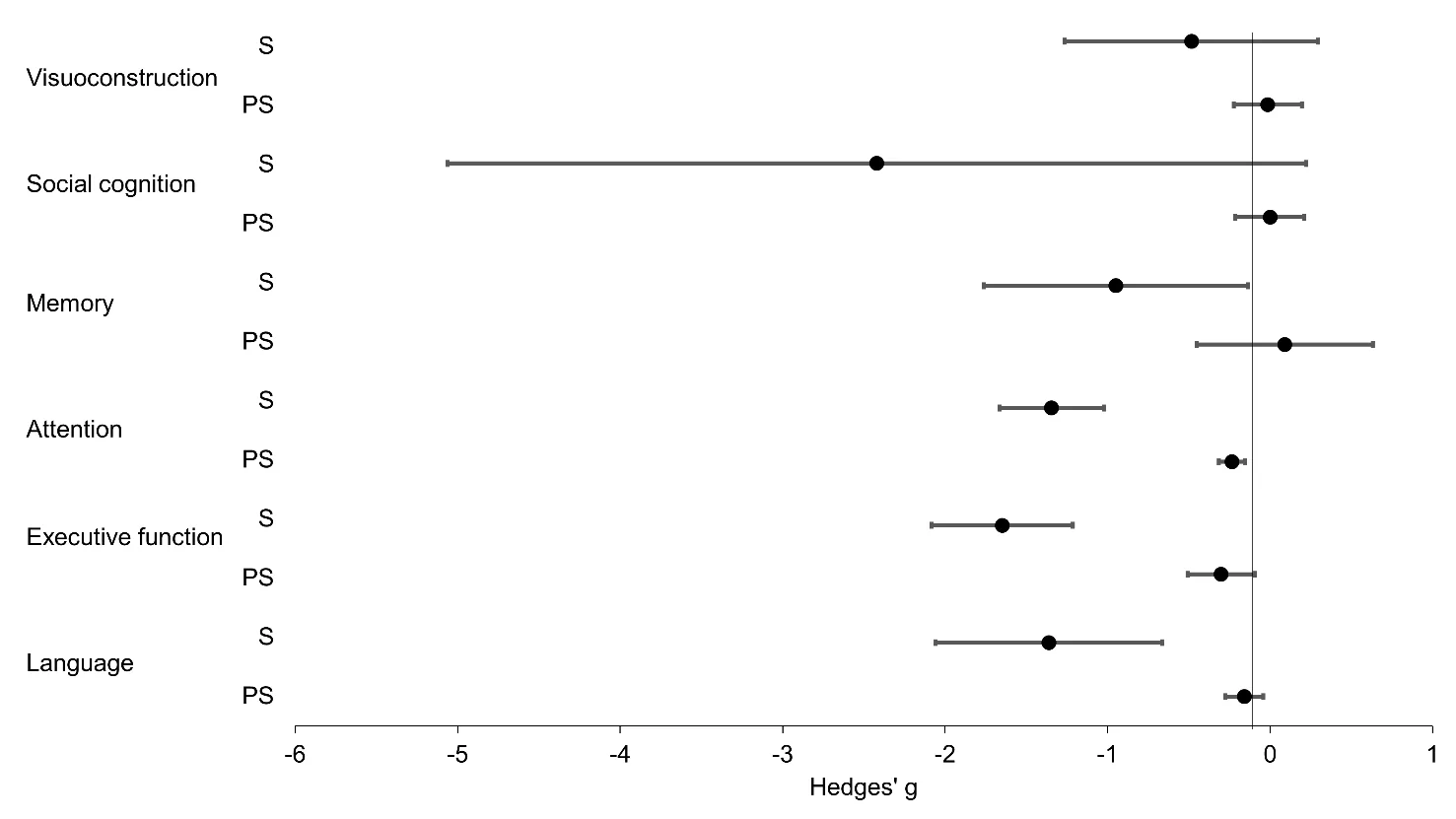
Forest plot illustrating overall effect sizes and bias-corrected 95% confidence intervals for each cognitive domain comparing presymptomatic and symptomatic *C9orf72* mutation carriers to controls.

Significant differences between presymptomatic mutation carriers and controls were found in the domains language, and attention and mental processing speed (Fig. 4). Language was assessed in seven studies, comprising 25 effect sizes, and a small effect of -0.16 (95% CI [-0.27, 0.04]; *t*(24) = -2.80, *p*=0.010) was found. There was no evidence for within-study or between-study variance (*p*>0.05). Attention and mental processing speed was assessed in four studies, comprising 19 effect sizes, and a small effect of -0.23 (95% CI [-0.32, -0.15]; *t*(18) = -6.06, *p*<0.001) was found. There was no evidence for within-study or between-study variance (p>0.05). No significant differences were found for executive function, memory, social cognition, and visuospatial functioning.

##### 3.1.4.2 The symptomatic stage

Significant differences compared to controls were found in the domains language, executive function, attention and mental processing speed, and memory (Fig. 4). Language was assessed in eight studies, comprising 23 effect sizes, and revealed a large effect of -1.36 (95% CI [-2.06, -0.66]; *t*(22) = -4.04, *p*<0.001). There was evidence for between-study variance (*p*<0.001), and moderator analyses revealed that effect sizes were larger for verbal fluency tests compared to semantic processing tests (*t*(20) = 2.45, *p*=0.024). There was also trend evidence that effect sizes were larger for verbal fluency tests compared to naming tests (*t*(20) = -1.87; *p*=0.076), and for naming tests compared to semantic processing tests (*t*(20) = -1.84; *p*=0.080). Executive function was assessed in seven studies, comprising 19 effect sizes, and a large effect of -1.67 (95% CI [-2.13, -1.21]; *t*(18) = -7.65, *p*<0.001) was found. There was evidence for within-study variance (*p* < 0.001), and moderator analysis revealed that mean education level (*t*(16) = -3.30, p=0.004) and subprocess were significant moderators. Effect sizes for inhibitory control tests were larger than for working memory tests (*t*(9) = -3.21, *p*<0.001) and cognitive flexibility tests (*t*(9) = -2.49, *p*=0.034). Attention and mental processing speed was assessed in five studies, comprising 12 effect sizes, and revealed a large effect of -1.35 (95% CI [-1.67, -1.02]; *t*(11) = -9.22, *p*<0.001). There was evidence for significant within-study variance (p<0.001), and moderator analysis demonstrated that effect sizes were larger for information processing tests than for attention tests (*t*(10) = -4.02, *p*=0.002). Memory was assessed in six studies, comprising 20 effect sizes, with a large effect of -0.95 (95% CI [-1.76, -0.13]; *t*(19) = -2.45, *p*=0.024). There was evidence for between-study variance (*p*<0.001), and moderator analysis revealed that effect sizes were larger for both immediate (*t*(17) = -3.35, *p*=0.004) and delayed (*t*(17) = -2.28, *p*=0.036) recall compared to cued recall. No significant differences were found for social cognition and visuospatial functioning.

#### 3.1.5 Publication bias

##### 3.1.5.1 Presymptomatic GRN mutation carriers

No indications for publication bias were found on any of the cognitive domains.

##### 3.1.5.2 Symptomatic GRN mutation carriers

Egger’s regression test indicated publication bias for the meta-analyses on differences between mutation carriers and controls in language (t(10) = -2.31, *p*=0.04), executive function (t(7) = -3.12, *p*=0.02), and memory (t(11) = -3.77, *p*<0.01).

##### 3.1.5.3 Presymptomatic MAPT mutation carriers

Egger’s regression test indicated publication bias for the meta-analyses on differences between mutation carriers and controls in language (t(21) = -3.21, *p*<0.01), executive function (t(17) = -4.33, *p*<0.01), attention (t(21) = -3.91, *p*<0.01), and memory (t(15) = -2.62, *p*=0.02),

##### 3.1.5.4 Symptomatic MAPT mutation carriers

Egger’s regression test indicated publication bias for the meta-analyses on differences between mutation carriers and controls in attention (t(11) = -2.73, *p*=0.02), and memory (t(19) -3.07, *p*<0.01).

##### 3.1.5.5. Presymptomatic C9of72 mutation carriers

Egger’s regression test indicated publication bias for the meta-analyses on differences between mutation carriers and controls in memory (t(19) = 11.20, *p*<0.01).

##### 3.1.5.6 Symptomatic C9orf72 mutation carriers

Egger’s regression test indicated publication bias for the meta-analyses on differences between mutation carriers and controls in language (t(21) = -2.28, *p*=0.03).

##### 3.1.5.7 Funnel plot asymmetry

Funnel plot asymmetry confirmed indications of publication bias (Fig. C.1-C.2), as described in Sections 3.1.5.1 - 3.1.5.7. However, in most cases publication bias was considered less likely as inspection of funnel plots (Fig. C.1-C.2) revealed that funnel asymmetry is probably due to some other reason (e.g., number of studies too small, heterogeneity between studies).

### 3.2 Systematic review

#### 3.2.1 Cognitive profile of TBK1 mutation carriers

Three case studies investigated neuropsychological performance in *TBK1* mutation carriers. Koriath et al. [35] and Yu et al. [36] report on two patients with bvFTD, finding deficits in naming, attention, executive function and memory. Single-word comprehension deficits and dyscalculia developed later in the disease course [35]. In the most recent study, Swift et al. [37] described a patient with nonfluent variant PPA, with progressive speech difficulties, while the other cognitive domains were intact apart from mild executive dysfunction. Next to symptoms characteristic for nonfluent variant PPA (i.c., effortful and non-fluent speech, phonemic errors, and agrammatism), the language profile was accompanied by binary reversals (i.e., saying yes when meaning no, and vice versa), difficulty in reading and writing, naming deficits, and impaired poly-syllabic single-word and sentence repetition, while semantic knowledge and single-word comprehension were spared.

#### 3.2.2 Cognitive profile of TARDBP mutation carriers

We identified two studies (*n*=31) that described neuropsychological changes in symptomatic *TARDBP* mutation carriers. Taking these two studies together, the majority of patients had bvFTD, while almost half of the patients also presented with linguistic deficits [38, 39]. Disorders in attention, executive function (e.g., abstract reasoning, initiation and conceptualization, planning), but also verbal and visual episodic memory were found, while visuospatial and -constructive abilities were spared. The language profile of patients with the *TARDBP* mutation was particularly characterized by visual and verbal semantic problems, including deficits in confrontation and picture naming, categorical fluency, word discrimination, single-word comprehension, and recognition and naming of famous faces.

#### 3.2.3 Cognitive profile of VCP mutation carriers

The heterogeneity in *VCP* disease is also reflected in its neuropsychological profile, as demonstrated by the three studies in symptomatic *VCP* mutation carriers [40]. Kalbe et al. [41] described two patients with progressive muscle weakness at baseline and 18-month follow-up, without overt dementia (global cognitive batteries, MMSE and DemTect, both above cut-off for dementia), but specific executive (i.c., shifting, interference, fluency) and social cognitive (i.c. theory of mind) dysfunction and decline. In the case study by Surampalli et al. [40] however, rapid cognitive impairment preceded any behavioural symptoms in one affected twin discordant for the *VCP* mutation, reflected in impairments on categorical fluency, confrontation naming, and concept shifting, and reduced performance on the same tests one year later, as well as impaired working memory. Lastly, the three patients described by Kim et al. [42] all presented with semantic variant PPA, as demonstrated by anomia, word comprehension deficits, semantic paraphasias, object and face agnosia, as well as visual memory and mental processing speed deficits.

#### 3.2.4 Cognitive profile of CHMP2B mutation carriers

We identified one study that described longitudinal neuropsychological changes over an 8-year time span in 17 presymptomatic *CHMP2B* mutation carriers [43]. Cross-sectional analyses demonstrated significant differences between mutation carriers and cognitively healthy controls with respect to visuospatial abilities and psychomotor speed, and trends towards significance in executive function (including working memory). No differences were found in memory and verbal fluency measures. Longitudinal analyses on a subset of presymptomatic mutation carriers showed significant decline with respect to psychomotor speed, executive function, and language (fluency).

#### 3.2.5 Cognitive profile of FUS mutation carriers

Two studies investigated the neuropsychological profile of symptomatic *FUS* mutation carriers. The studies by Lee et al. [44] (n=1) and Rohrer et al. [45] (n=5) demonstrated the most prominent deficits to be in the memory and executive function domain, while performance in other cognitive domains - attention and mental processing speed, language (naming) and visuospatial abilities - was within the normal range upon initial neuropsychological assessment. The study by Lee et al. [44] additionally included comprehensive social cognition testing, showing deficits in amongst others emotion recognition, decision-making, higher-order theory of mind tasks, empathy and social awareness.

## 4. Discussion

We examined cognitive profiles across mutations and clinical disease stages of familial FTLD. Twenty-seven and 11 studies were eligible for the meta-analysis and systematic review, respectively. By pooling all the available cognitive data in a rare disorder as FTLD we were able to provide a weighted estimate of the magnitude of effects, while the majority of studies into neuropsychological deficits in familial FTLD only included small case- or family-based studies. Cognitive decline in language, and attention and mental processing speed was already present in the presymptomatic stage of *C9orf72* mutation carriers, and in executive function in *GRN* mutation carriers, albeit to a lesser extent than in the symptomatic stage. In the domains language and executive function, cognitive sub-processes were differentially affected across mutations. This study has increased our knowledge of cognitive deficits and more specifically the affected cognitive subprocesses accompanying their mutations.

*GRN* mutation carriers already showed minor deficits in executive function in the presymptomatic stage. Executive function was also the most affected domain in the symptomatic stage, followed by language, memory, and attention and mental processing speed. Previous studies have shown that *GRN* mutation carriers have more executive function problems and fewer social cognitive and behavioral disturbances than other TDP-43 positive patients [46, 47]. This corresponds with the findings in our study, as no deficits in social cognition were found. Interestingly, cognitive flexibility was more affected than working memory in symptomatic *GRN* mutation carriers. This is most likely related to the fact that the studies included in our meta-analysis used digit span backwards as a measure of working memory. A previous study reported that while non-verbal memory is affected in *GRN*-FTD, verbal working memory is relatively preserved, which is thought to be related to reduced glucose metabolism in the right frontal cortex, a brain structure that is involved in spatial working memory [20]. Moreover, information processing speed was more affected than attention in symptomatic *GRN* mutation carriers. This could be explained by the fact that clinical presentations of *GRN* mutations also include movement disorders with extrapyramidal features and the sub-process information processing was only composed of tests with a strong motor component, while the sub-process attention was not. Alternatively, deficits in information processing speed could be related to white matter hyperintensities - which are specifically seen in *GRN* mutations, and not in other mutations [48]. Interestingly, we found significant between-study variance for the language domain that could not be explained by demographic variables or cognitive subprocess. A potential explanation for this could be the difference between studies in the distribution of included phenotypes. For example, Olney et al. (2020) [67] included only bvFTD cases, whereas in both Moore et al. (2020) [18] and Russell (2022) [22] the majority of patients had a PPA diagnosis.

In *MAPT* mutations, significant decline in the domain’s language, executive function, attention and mental processing speed, and memory was found in symptomatic mutation carriers, but not yet in presymptomatic mutation carriers. Together with memory, the language domain is the most affected in symptomatic mutation carriers. In the language domain, naming and semantic processing are more compromised than verbal fluency. Naming and semantic impairments are well-described in *MAPT*-FTD [18, 49]. Similar to svPPA, early atrophy of the anterior temporal lobe is found in *MAPT* mutations, which is thought to be the underpinning of semantic memory functioning [50]. In contrast to our results, naming has been found to be affected in the presymptomatic phase [13, 51]. Most likely the small sample size of presymptomatic *MAPT* mutation carriers and the fact that naming was not assessed in half of the studies are a possible explanation for this. In contrast to *GRN* mutation carriers, effect sizes were larger for working memory than for cognitive flexibility in symptomatic *MAPT* mutation carriers. The strong language component of the verbal memory working tests included in our study could provide a possible explanation of this finding. Memory as the most affected cognitive domain in *MAPT*-FTD is not a surprising finding, given the atrophy often found in the mesial temporal lobe, in particular the hippocampus, regions known to be involved in memory [52]. It is of note that there were no significant differences in the sub-processes within the memory domain. One previous study found deficits in both immediate and delayed verbal recall in the presymptomatic stage [17], while a visual memory test showed delayed recall to be earlier affected than figure copy and recognition [53]. It can be hypothesized that *MAPT*-FTD is characterized by anterograde amnesia as the result of the mesial temporal lobe (hippocampal) atrophy, as is for instance the case in Alzheimer’s dementia, and therefore both immediate and delayed recall, *and* recognition are affected. Alternatively, the sample size of *MAPT* mutation carriers was too small, and therefore lacking statistical power, to reliably assess significant differences in the underlying memory sub-processes. Surprisingly, significant within-study variance was found for the attention and memory domains, with the latter also being affected by between-study variance. This was not moderated by demographic variables or cognitive subprocess. In contrast with *GRN* mutation carriers, it is less likely that the distribution of clinical phenotypes in the included *MAPT* studies explains this, as nearly 97% of cases were diagnosed with bvFTD. Another explanation could be the heterogeneity between and within studies to measure specific cognitive subprocesses. For example, some studies administered digit span forward to measure attention, whereas others administered TMT - A or Stroop color-word cards I and/or II. Similarly, for the memory domain some studies administered both or only tests for verbal and visual episodic memory, which could be differentially affected in genetic variants of FTD [17, 53].

In *C9orf72*, language, attention and mental processing speed were affected in both presymptomatic as well as symptomatic mutation carriers. Memory and executive function were only involved in symptomatic mutation carriers. This suggests that minor, multi-domain cognitive deficits are already present in the presymptomatic stage, but worsen over time as mutation carriers convert to the symptomatic stage, in which executive function and memory deficits appear. In line with our findings, a proportion of patients with *C9orf72* repeat expansions have a long disease course, starting in early adulthood, with minor to no clinical or neuroimaging deterioration [54], suggestive of a neurodevelopmental origin [55]. In fact, as brain atrophy and white matter integrity loss are found to be present several decades before overt onset of dementia, the presymptomatic phase is most likely spanning a long time period, with a superimposed neurodegenerative process later in life [13, 56, 57]. The involvement of multiple cognitive domains coincides with the wide range of presenting clinical subtypes and diffuse cortical and subcortical atrophy that characterize this mutation [58]. In the executive domain inhibitory control was more affected than cognitive flexibility and working memory. This sets the *C9orf72* mutation carriers apart from both *GRN* and *MAPT* mutation carriers. Moreover, verbal fluency was more compromised than semantic processing and naming in symptomatic mutation carriers. Impairments in verbal fluency can be the result of the cerebellar atrophy often found in *C9orf72*-FTD, as this subcortical region plays a crucial role in motor performance and executive processes necessary for organizing and monitoring word output [59]. The more subcortical character of *C9orf72*-FTD is also reflected in the fact that in our study information processing is affected, and cued recall is better than immediate and delayed recall in the memory domain (suggestive of a retrieval problem and not a consolidation problem).

Strikingly, no significant effect sizes were found for social cognition in any of the genetic subgroups. This is a surprising finding given that deficits in social cognition are considered to be at the heart of problems in behavior and personality seen in individuals with FTD (e.g., lack of empathy/sympathy, inappropriate behavior), and are often the first symptoms to be recognized by family members and friends [60]. Interestingly, the effect sizes for social cognition in symptomatic mutation carriers were the largest compared to other cognitive domains, particularly in *GRN* and *C9orf72* mutation carriers. A lack of statistical power could potentially explain why these effect sizes were not significant. In total, only seven studies assessed social cognition, of which three included symptomatic *C9orf72* mutation carriers, but only one included symptomatic *GRN* and *MAPT* mutation carriers. Although there are no strict guidelines regarding the number of effect sizes necessary to perform a meta-analysis, it is recommended to include at least five to ten studies to obtain a sufficiently robust and reliable analysis [61]. As deficits in social cognition are considered the hallmark of FTD, one could expect deficits in this domain already being present in the presymptomatic stage. Potential explanations as to why we did not find these deficits could be a lack of standardized instruments for social cognition assessment and robust norms across cultures/countries, and the lack of an empirically supported definition of social cognition [62].

Case studies in carriers with mutations in the *TBK1, TARDBP, VCP, CHMP2B*, and *FUS* genes show a divergent cognitive profile. While most mutations are associated with bvFTD as clinical phenotype, a majority of patients had a PPA diagnosis, and evident linguistic deficits were described, in addition to deficits in amongst others attention, executive function, and memory. The study by Stokholm et al. [43] was the only one describing presymptomatic (*CHMP2B*) mutation carriers, showing deficits in visuospatial abilities and psychomotor speed, and to a lesser degree deficits in executive function, while memory and language were not affected. More research is needed to determine if there are already cognitive deficits present in the presymptomatic stage of the other genetic groups. Confirming the underrepresented nature of this domain in cognitive studies, the study by Lee et al. [44] in *FUS* mutation carriers was the only one included in this systematic review that investigated deficits in social cognition. Pooling of patients with rarer FTLD mutations will form a more comprehensive picture of the cognitive profiles in genetic FTLD.

The major strength of our study is the use of a combined meta-analytic and systematic review approach, which gives a comprehensive overview of the cognitive profiles in eight different genetic groups of familial FTLD. Furthermore, the application of a three-level mixed effects model to account for interdependency of effect sizes within studies allowed us to include all effect sizes reported within studies thereby preserving all relevant information and achieving maximum statistical power. Not only did we take the large cognitive domains into account, but we also investigated specific sub-processes, giving us additional information about the cognitive profile in the different genetic groups, and heterogeneity between different study findings. Lastly, our analysis not only focused on the three major genetic variants but also encompassed the rarer mutations associated with FTLD. To date, the available literature mainly consists of case studies or case series, and by systematically reviewing these we have found that cognition is differentially affected in these subtypes as well. This underlines the importance of pooling rarer cases in multi-center cohort studies, potentially allowing for quantitative analysis of cognitive data in the future. A limitation of our study is the potential heterogeneity of the patient samples with respect to underlying mutation, clinical phenotype, and disease duration/time to symptom onset [5, 6-65]. A drawback as a result of the rarity of genetic FTLD was the small sample size in the *MAPT* studies were low. This could potentially also have led to funnel plot asymmetry indicating publication/selection bias [66].

To conclude, we found partly unique, partly overlapping cognitive profiles depending on the underlying FTLD mutation. In most mutations, cognitive decline was already present in the presymptomatic stage, albeit to a lesser extent than in the symptomatic stage. In the domains language and executive function, gene-specific cognitive sub-processes were affected in different mutations. The latter suggests that future studies should - in addition to analyzing only the major cognitive domains - also look into sub-processes, as they provide additional information about the unique cognitive ‘fingerprint’ accompanying FTLD mutations, which can aid us in differential diagnosis and the selection of clinical endpoints for upcoming disease-modifying trials.

## Supplementary Materials

The Supplementary data can be found online at: www.aginganddisease.org/EN/10.14336/AD.2024.0183.
