# Supplementary material for: Neuropsychological Profiles in Genetic Frontotemporal Dementia: A Meta-Analysis and Systematic Review
Source: Aging Dis. 2024 Jun 24;16(3):1378–96. doi: 10.14336/AD.2024.0183 (PMC12096942; doi:10.14336/AD.2024.0183)
Supplement: Supplementary file 1 — The Supplementary data can be found online at: www.aginganddisease.org/EN/10.14336/AD.2024.0183. [file AD-16-3-1378-s.pdf]

Profiles in Genetic Frontotemporal Dementia: A Meta-Analysis and Systematic Review

Jackie M. Poos, Esther van den Berg, Liset de Boer, Sabrina Meertens-Gunput, Elise G.P. Dopper, Harro Seelaar, Lize C. Jiskoot

© 2024. Poos JM. et al. Published online at http://www.aginganddisease.org/EN/10.14336/AD.2024.0183

SUPPLEMENTARY DATA

Appendix A. Search terms used for this meta-analysis.

Database searched	Platform	Years of coverage	Records	Records after duplicates removed
Embase	Embase.com	1971 - Present	2179	2159
Medline ALL	Ovid	1946 - Present	1286	243
Web of Science Core Collection*	Web of Knowledge	1975 - Present	2053	833
PsycINFO	Ovid	1806 - Present	906	195
Cochrane Central Register of Controlled Trials	Wiley	1992 - Present	18	4
Additional Search Engines: Google Scholar			200	100
Total			6642	3534

*Science Citation Index Expanded (1975-present); Social Sciences Citation Index (1975-present); Arts & Humanities Citation Index (1975-present); Conference Proceedings Citation Index- Science (1990-present); Conference Proceedings Citation Index - Social Science & Humanities (1990-present); Emerging Sources Citation Index (2005-present).

Embase.com

('frontotemporal dementia'/exp OR 'Pick presenile dementia'/de OR 'tauopathy'/de OR (((frontotemporal* OR fronto-temporal*) NEAR/3 (dementia* OR degenerat*)) OR tauopath* OR FTD OR FTLD OR (pick* NEAR/3 disease*) OR progressive-aphasia*):ab,ti,kw) AND ('genetics'/exp OR 'heredity'/exp OR 'progranulin'/exp OR 'valosin containing protein'/de OR (heredit* OR famil* OR genet* OR MAPT OR microtubule-associated-protein-tau* OR GRN OR progranulin* OR granulin* OR C9ORF72 OR Chromosome-9-open-reading-frame* OR TBK1 OR tank-binding-kinase-1* OR FUS OR fused-in-sarcoma* OR VCP OR valosin-containing-protein* OR CHMP2B OR charged-multivesicular-body-protein-2B):ab,ti,kw) AND ('cognition'/de OR 'attention'/de OR 'executive function'/de OR 'memory'/exp OR 'memory disorder'/de OR 'social cognition'/de OR 'neuropsychological test'/de OR 'language'/de OR (cognit* OR attention* OR ((executive*) NEAR/3 (function* OR control*)) OR memor* OR neuropsychological* OR language*):ab,ti,kw) NOT ((animal/exp OR animal*:de OR nonhuman/de) NOT ('human'/exp)) NOT [review]/lim NOT [conference abstract]/lim

Medline

(exp Frontotemporal Dementia/ OR Tauopathies/ OR (((frontotemporal* OR fronto-temporal*) ADJ3 (dementia* OR degenerat*)) OR tauopath* OR FTD OR FTLD OR (pick* ADJ3 disease*) OR progressive-aphasia*).ab,ti,kf.) AND (exp Genetics/ OR Heredity/ OR exp Progranulins/ OR Valosin Containing Protein/ OR (heredit* OR famil* OR genet* OR MAPT OR microtubule-associated-protein-tau* OR GRN OR progranulin* OR granulin* OR C9ORF72 OR Chromosome-9-open-reading-frame* OR TBK1 OR tank-binding-kinase-1* OR FUS OR fused-in-sarcoma* OR VCP OR valosin-containing-protein* OR CHMP2B OR charged-multivesicular-body-protein-2B).ab,ti,kf.) AND (Cognition/ OR Attention/ OR Executive Function/ OR exp Memory/ OR Memory Disorders/ OR Neuropsychological Tests/ OR Language/ OR (cognit* OR attention* OR ((executive*) ADJ3 (function* OR control*)) OR memor* OR neuropsychological* OR language*).ab,ti,kf.) NOT (exp animals/ NOT humans/) NOT (news OR comment* OR editorial* OR congres* OR abstract* OR book* OR chapter* OR dissertation abstract* OR review*).pt.

Web of Science

TS=(((((frontotemporal* OR fronto-temporal*) NEAR/2 (dementia* OR degenerat*)) OR tauopath* OR FTD OR FTLD OR (pick* NEAR/2 disease*) OR progressive-aphasia*)) AND ((heredit* OR famil* OR genet* OR MAPT OR microtubule-associated-protein-tau* OR GRN OR progranulin* OR granulin* OR C9ORF72 OR

Chromosome-9-open-reading-frame* OR TBK1 OR tank-binding-kinase-1* OR FUS OR fused-in-sarcoma* OR VCP OR valosin-containing-protein* OR CHMP2B OR charged-multivesicular-body-protein-2B)) AND ((cognit* OR attention* OR ((executive*) NEAR/2 (function* OR control*)) OR memor* OR neuropsychological* OR language*)) NOT ((animal* OR rat OR rats OR mouse OR mice OR murine OR dog OR dogs OR canine OR cat OR cats OR feline OR rabbit OR cow OR cows OR bovine OR rodent* OR sheep OR ovine OR pig OR swine OR porcine OR veterinar* OR chick* OR zebrafish* OR baboon* OR nonhuman* OR primate* OR cattle* OR goose OR geese OR duck OR macaque* OR avian* OR bird* OR fish*) NOT (human* OR patient* OR women OR woman OR men OR man))) AND DT=(Article OR Review OR Letter OR Early Access)

Cochrane

((((frontotemporal* OR fronto NEXT temporal*) NEAR/3 (dementia* OR degenerat*)) OR tauopath* OR FTD OR FTLD OR (pick* NEAR/3 disease*) OR progressive NEXT aphasia*):ab,ti,kw) AND ((heredit* OR famil* OR genet* OR MAPT OR microtubule NEXT associated NEXT protein NEXT tau* OR GRN OR progranulin* OR granulin* OR C9ORF72 OR "Chromosome 9 open reading frame" OR TBK1 OR tank NEXT binding NEXT kinase NEXT 1* OR FUS OR fused NEXT in NEXT sarcoma* OR VCP OR valosin NEXT containing NEXT protein* OR CHMP2B OR charged NEXT multivesicular NEXT body NEXT protein NEXT 2B):ab,ti,kw) AND ((cognit* OR attention* OR ((executive*) NEAR/3 (function* OR control*)) OR memor* OR neuropsychological* OR language*):ab,ti,kw) NOT "conference abstract":pt

PsycINFO

(Semantic Dementia/ OR Picks Disease/ OR (((frontotemporal* OR fronto-temporal*) ADJ3 (dementia* OR degenerat*)) OR tauopath* OR FTD OR FTLD OR (pick* ADJ3 disease*) OR progressive-aphasia*).ab,ti.) AND (exp Genetics/ OR (heredit* OR famil* OR genet* OR MAPT OR microtubule-associated-protein-tau* OR GRN OR progranulin* OR granulin* OR C9ORF72 OR Chromosome-9-open-reading-frame* OR TBK1 OR tank-binding-kinase-1* OR FUS OR fused-in-sarcoma* OR VCP OR valosin-containing-protein* OR CHMP2B OR charged-multivesicular-body-protein-2B).ab,ti.) AND (Cognition/ OR Attention/ OR Executive Function/ OR exp Memory/ OR Memory Disorders/ OR Social Cognition/ OR Neuropsychological Assessment/ OR Language/ OR (cognit* OR attention* OR ((executive*) ADJ3 (function* OR control*)) OR memor* OR neuropsychological* OR language*).ab,ti.) NOT ((animal.po. OR exp animals/) NOT human.po.) NOT (news OR comment* OR editorial* OR congres* OR abstract* OR book* OR chapter* OR dissertation abstract* OR review*).pt.

Google Scholar

'frontotemporal|fronto-temporal dementia|degeneration'|tauopathy|tauopathies|FTD|FTLD|'pick|picks disease' hereditary|familial|genetic|genetics cognition|attention|'executive function|control'|memory|neuropsychological|language

Appendix B. Overview of cognitive domains and processes, and tests assigned in meta-analysis.

Cognitive domain	Cognitive process	Tests
Language	Naming	(abbreviated) BNT, MINT, graded naming test
	Fluency	Semantic and letter fluency tests
	Semantic processing	Modified Camel & Cactus Test, SAT verbal, Pyramids & Palm trees
Attention & mental processing speed	Attention	TMT A, Digit Span forwards, Stroop CWT card 1 and 2, Spatial Span forwards
	Information processing	LDST, Digit Symbol test
Executive function	Working memory	Digit Span backwards, Spatial Span backwards, Letter Number Sequencing
	Inhibitory control	Stroop CWT card 3, Hayling test

© 2024. Poos JM. et al. Published online at http://www.aginganddisease.org/EN/10.14336/AD.2024.0183

	Cognitive flexibility	TMT B, (m)WCST, design fluency, Brixton test, D-KEFS sorting test
Memory	Immediate recall	RAVLT immediate recall, RMBT immediate recall, CERAD word list immediate recall, logical memory immediate recall, Craft immediate recall, FCSRT immediate recall, CVLT immediate recall, SRT immediate recall
	Delayed free recall	Benson Figure recall, RAVLT delayed recall, RMBT delayed recall, CERAD word list delayed recall, RCF delayed recall, logical memory delayed recall, Craft delayed recall, FCSRT delayed recall, CVLT free recall, short story recall, SRT delayed recall, Visual Reproduction recall
	Cued free recall	Benson Figure recognition, RAVLT recognition, VAT, CVLT cued recall, CERAD word list recognition, Doors B
Social cognition	Perception and automatic attribution	ERT, facial emotion recognition, Ekman 60 faces, mini-SEA emotion recognition
	Understanding and Interpretation	Faux-pas, Happé cartoons, mini-SEA faux-pas
	Reasoning and Regulation	SNQ
Visuospatial abilities	Visuoconstruction	Block design, Benson Figure copy, clock drawing, RCF copy, CERAD constructional praxis, Visual Reproduction

Abbreviations: BNT, Boston Naming Test; MINT, Multilingual Naming Test; SAT, Semantic Association Test; TMT, Trail Making Test; CWT, colour word test; LDST, Letter Digit Substitution Test; (m)WCST, (modified) Wisconsin Card Sorting Test; D-KEFS, Delis-Kaplan Executive Function System; RAVLT, Rey Auditory Verbal Learning Test; RBMT; Rivermead Behavioural Memory Test; CERAD, Consortium to Establish a Registry for Alzheimer's Disease; FCSRT, Free and Cued Selective Reminding Test; CVLT, California Verbal Learning Test; SRT, Selective Reminding Test; RCF, Rey-Osterrieth Complex Figure; ERT, Emotion Recognition Test; mini-SEA, mini Social Cognition & Emotional Assessment; SNQ, Social Norms Questionnaire.

© 2024. Poos JM. et al. Published online at http://www.aginganddisease.org/EN/10.14336/AD.2024.0183

Appendix C. Figures

Fig C.1 Funnel plot for attention, executive function, and memory in presymptomatic and symptomatic mutation carriers.

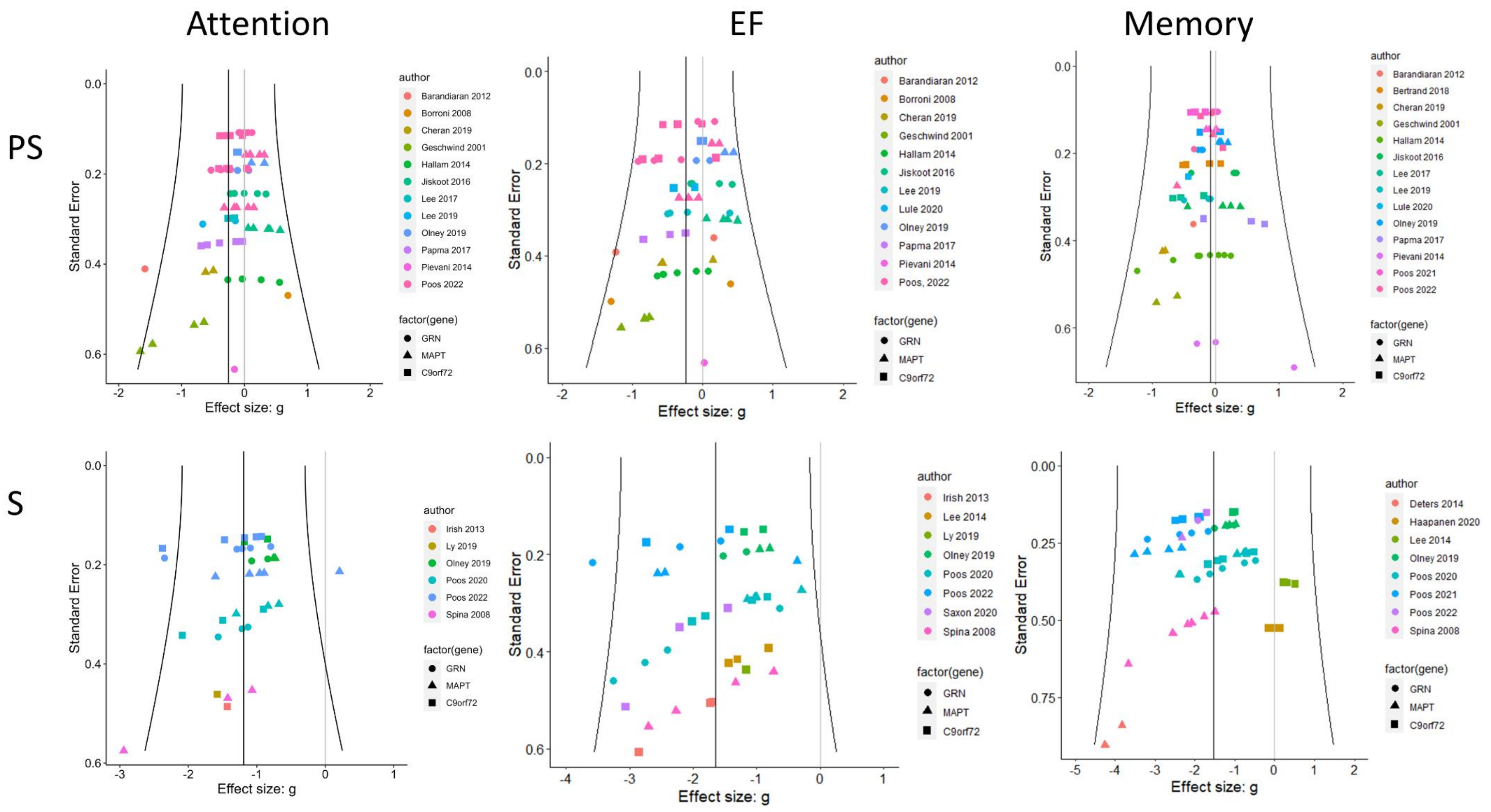

© 2024. Poos JM. et al. Published online at http://www.aginganddisease.org/EN/10.14336/AD.2024.0183

Figure C.2. Funnel plot for language, social cognition, and visuoconstruction in presymptomatic and symptomatic mutation carriers.

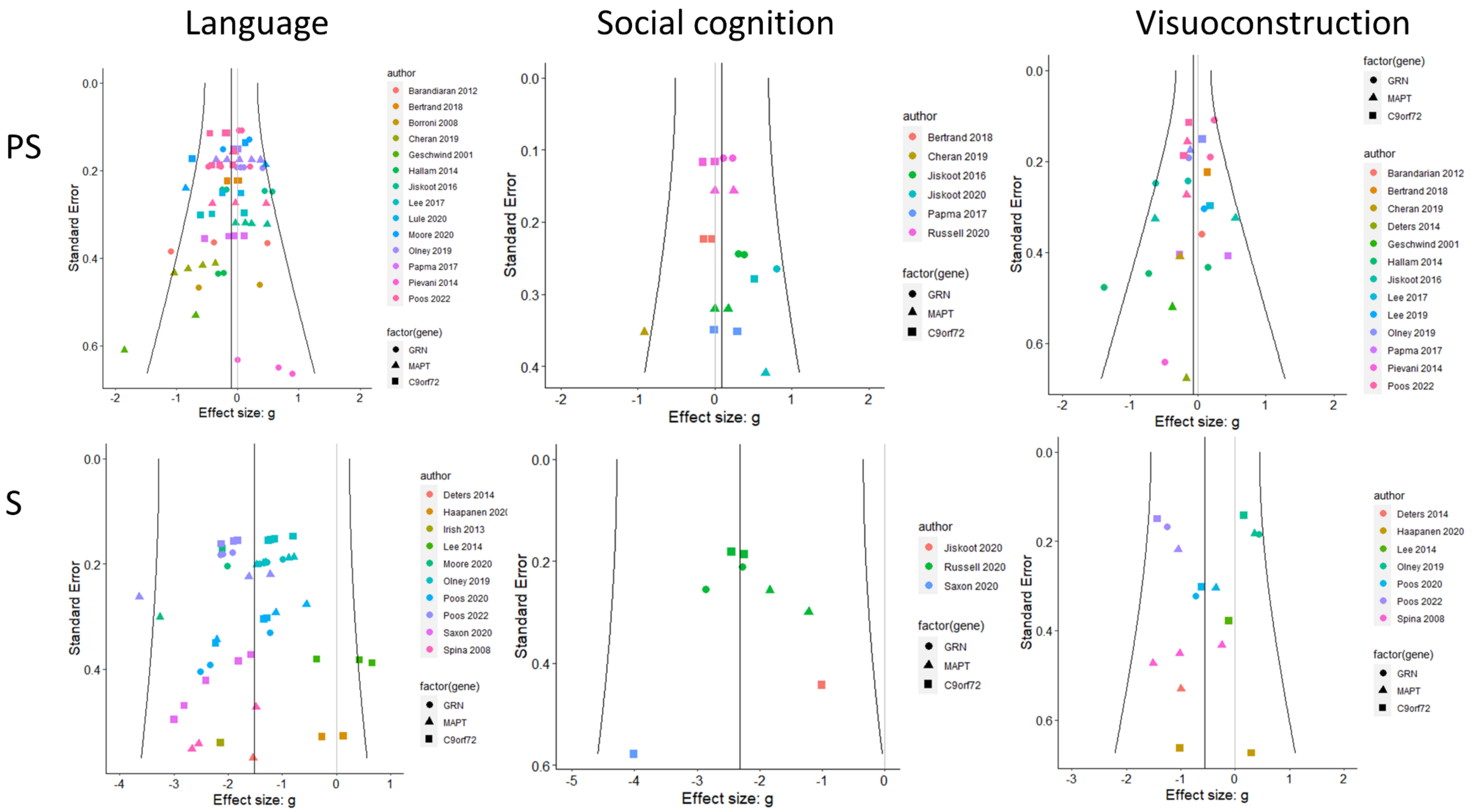

© 2024. Poos JM. et al. Published online at http://www.aginganddisease.org/EN/10.14336/AD.2024.0183

SUPPLEMENTARY DATA

Appendix D. Overview of the sample sizes included in the meta-analysis for each cognitive domain and sub-process.

Domain	Subprocess	*n* mutation carriers		*n* controls
Language	Total domain	Total sample	3613	2001
		Total *GRN*	1114	
		Total *MAPT*	850	
		Total *C9orf72*	1619	
		Total presymptomatic	2132	
		Presymptomatic *GRN*	817	
		Presymptomatic *MAPT*	589	
		Presymptomatic *C9orf72*	841	
		Total symptomatic	1336	
		Symptomatic *GRN*	297	
		Symptomatic *MAPT*	261	
		Symptomatic *C9orf72*	778	
	Naming	Total sample	1253	597
		Total *GRN*	379	
		Total *MAPT*	252	
		Total *C9orf72*	622	
		Total presymptomatic	706	
		Presymptomatic *GRN*	270	
		Presymptomatic *MAPT*	145	
		Presymptomatic *C9orf72*	291	
		Total symptomatic	547	
		Symptomatic *GRN*	109	
		Symptomatic *MAPT*	107	
		Symptomatic *C9orf72*	331	
	Fluency	Total sample	1445	
		Total *GRN*	524	
		Total *MAPT*	260	
		Total *C9orf72*	661	

© 2024. Poos JM. et al. Published online at http://www.aginganddisease.org/EN/10.14336/AD.2024.0183

		Total presymptomatic	728	785
		Presymptomatic *GRN*	415	
		Presymptomatic *MAPT*	145	
		Presymptomatic *C9orf72*	313	
		Total symptomatic	572	
		Symptomatic *GRN*	109	
		Symptomatic *MAPT*	115	
		Symptomatic *C9orf72*	348	
	Semantic processing	Total sample	915	619
		Total *GRN*	211	
		Total *MAPT*	338	
		Total *C9orf72*	336	
		Total presymptomatic	698	
		Presymptomatic *GRN*	132	
		Presymptomatic *MAPT*	299	
		Presymptomatic *C9orf72*	237	
		Total symptomatic	217	
		Symptomatic *GRN*	79	
		Symptomatic *MAPT*	39	
		Symptomatic *C9orf72*	99	
Attention & mental processing speed	Total domain	Total sample	3684	1437
		Total *GRN*	1287	
		Total *MAPT*	676	
		Total *C9orf72*	1721	
		Total presymptomatic	2282	
		Presymptomatic *GRN*	1049	
		Presymptomatic *MAPT*	400	
		Presymptomatic *C9orf72*	833	
		Total symptomatic	1494	
		Symptomatic *GRN*	330	

© 2024. Poos JM. et al. Published online at http://www.aginganddisease.org/EN/10.14336/AD.2024.0183

		Symptomatic *MAPT*	276	
		Symptomatic *C9orf72*	888	
	Attention	Total sample	3043	1041
		Total *GRN*	1043	
		Total *MAPT*	579	
		Total *C9orf72*	1421	
		Total presymptomatic	1760	
		Presymptomatic *GRN*	759	
		Presymptomatic *MAPT*	327	
		Presymptomatic *C9orf72*	674	
		Total symptomatic	1283	
		Symptomatic *GRN*	284	
		Symptomatic *MAPT*	252	
		Symptomatic *C9orf72*	747	
	Information processing	Total sample	641	396
		Total *GRN*	244	
		Total *MAPT*	97	
		Total *C9orf72*	300	
		Total presymptomatic	522	
		Presymptomatic *GRN*	290	
		Presymptomatic *MAPT*	73	
		Presymptomatic *C9orf72*	159	
		Total symptomatic	211	
		Symptomatic *GRN*	46	
		Symptomatic *MAPT*	24	
		Symptomatic *C9orf72*	141	
Executive function	Total domain	Total sample	3568	
		Total *GRN*	1123	
		Total *MAPT*	724	
		Total *C9orf72*	1721	

© 2024. Poos JM. et al. Published online at http://www.aginganddisease.org/EN/10.14336/AD.2024.0183

		Total presymptomatic	2000	1945
		Presymptomatic *GRN*	819	
		Presymptomatic *MAPT*	410	
		Presymptomatic *C9orf72*	771	
		Total symptomatic	1568	
		Symptomatic *GRN*	304	
		Symptomatic *MAPT*	314	
		Symptomatic *C9orf72*	950	
	Working memory	Total sample	1178	630
		Total *GRN*	376	
		Total *MAPT*	252	
		Total *C9orf72*	550	
		Total presymptomatic	662	
		Presymptomatic *GRN*	267	
		Presymptomatic *MAPT*	145	
		Presymptomatic *C9orf72*	250	
		Total symptomatic	516	
		Symptomatic *GRN*	109	
		Symptomatic *MAPT*	107	
		Symptomatic *C9orf72*	300	
	Inhibitory control	Total sample	861	501
		Total *GRN*	281	
		Total *MAPT*	147	
		Total *C9orf72*	433	
		Total presymptomatic	512	
		Presymptomatic *GRN*	215	
		Presymptomatic *MAPT*	85	
		Presymptomatic *C9orf72*	212	
		Total symptomatic	349	
		Symptomatic *GRN*	66	

© 2024. Poos JM. et al. Published online at http://www.aginganddisease.org/EN/10.14336/AD.2024.0183

		Symptomatic *MAPT*	62	
		Symptomatic *C9orf72*	221	
	Cognitive flexibility	Total sample	1529	814
		Total *GRN*	466	
		Total *MAPT*	325	
		Total *C9orf72*	738	
		Total presymptomatic	826	
		Presymptomatic *GRN*	337	
		Presymptomatic *MAPT*	180	
		Presymptomatic *C9orf72*	309	
		Total symptomatic	703	
		Symptomatic *GRN*	129	
		Symptomatic *MAPT*	145	
		Symptomatic *C9orf72*	429	
Memory	Total domain	Total sample	6820	2064
		Total *GRN*	2049	
		Total *MAPT*	1487	
		Total *C9orf72*	3284	
		Total presymptomatic	3853	
		Presymptomatic *GRN*	1398	
		Presymptomatic *MAPT*	846	
		Presymptomatic *C9orf72*	1609	
		Total symptomatic	2967	
		Symptomatic *GRN*	651	
		Symptomatic *MAPT*	641	
		Symptomatic *C9orf72*	1675	
	Immediate recall	Total sample	1658	
		Total *GRN*	487	
		Total *MAPT*	379	
		Total *C9orf72*	792	

		Total presymptomatic	863	694
		Presymptomatic *GRN*	315	
		Presymptomatic *MAPT*	181	
		Presymptomatic *C9orf72*	367	
		Total symptomatic	795	
		Symptomatic *GRN*	172	
		Symptomatic *MAPT*	198	
		Symptomatic *C9orf72*	425	
	Delayed free recall	Total sample	2692	694
		Total *GRN*	781	
		Total *MAPT*	592	
		Total *C9orf72*	1319	
		Total presymptomatic	1509	
		Presymptomatic *GRN*	520	
		Presymptomatic *MAPT*	349	
		Presymptomatic *C9orf72*	640	
		Total symptomatic	1183	
		Symptomatic *GRN*	261	
		Symptomatic *MAPT*	243	
		Symptomatic *C9orf72*	679	
	Cued free recall	Total sample	2470	676
		Total *GRN*	781	
		Total *MAPT*	516	
		Total *C9orf72*	1173	
		Total presymptomatic	1481	
		Presymptomatic *GRN*	563	
		Presymptomatic *MAPT*	316	
		Presymptomatic *C9orf72*	602	
		Total symptomatic	989	
		Symptomatic *GRN*	218	

© 2024. Poos JM. et al. Published online at http://www.aginganddisease.org/EN/10.14336/AD.2024.0183

		Symptomatic *MAPT*	200	
		Symptomatic *C9orf72*	571	
Social cognition	Total domain	Total sample	1416	1044
		Total *GRN*	585	
		Total *MAPT*	258	
		Total *C9orf72*	573	
		Total presymptomatic	950	
		Presymptomatic *GRN*	475	
		Presymptomatic *MAPT*	198	
		Presymptomatic *C9orf72*	277	
		Total symptomatic	466	
		Symptomatic *GRN*	110	
		Symptomatic *MAPT*	60	
		Symptomatic *C9orf72*	296	
	Perception and automatic attribution	Total sample	877	673
		Total *GRN*	391	
		Total *MAPT*	166	
		Total *C9orf72*	320	
		Total presymptomatic	514	
		Presymptomatic *GRN*	313	
		Presymptomatic *MAPT*	124	
		Presymptomatic *C9orf72*	77	
		Total symptomatic	363	
		Symptomatic *GRN*	78	
		Symptomatic *MAPT*	42	
		Symptomatic *C9orf72*	243	
	Understanding and Interpretation	Total sample	527	
		Total *GRN*	194	
		Total *MAPT*	80	
		Total *C9orf72*	253	

© 2024. Poos JM. et al. Published online at http://www.aginganddisease.org/EN/10.14336/AD.2024.0183

		Total presymptomatic	424	339
		Presymptomatic *GRN*	162	
		Presymptomatic *MAPT*	62	
		Presymptomatic *C9orf72*	200	
		Total symptomatic	103	
		Symptomatic *GRN*	32	
		Symptomatic *MAPT*	18	
		Symptomatic *C9orf72*	53	
	Reasoning and Regulation	Total sample	12	32
		Total *GRN*	0	
		Total *MAPT*	12	
		Total *C9orf72*	0	
		Total presymptomatic	12	
		Presymptomatic *GRN*	0	
		Presymptomatic *MAPT*	12	
		Presymptomatic *C9orf72*	0	
		Total symptomatic	0	
		Symptomatic *GRN*	0	
		Symptomatic *MAPT*	0	
		Symptomatic *C9orf72*	0	
Visuospatial abilities	Total domain	Total sample	1299	740
		Total *GRN*	417	
		Total *MAPT*	280	
		Total *C9orf72*	602	
		Total presymptomatic	775	
		Presymptomatic *GRN*	308	
		Presymptomatic *MAPT*	158	
		Presymptomatic *C9orf72*	309	
		Total symptomatic	524	
		Symptomatic *GRN*	109	

© 2024. Poos JM. et al. Published online at http://www.aginganddisease.org/EN/10.14336/AD.2024.0183

		Symptomatic *MAPT*	122	
		Symptomatic *C9orf72*	293	
	Visuoconstruction	Total sample	1299	740
		Total *GRN*	417	
		Total *MAPT*	280	
		Total *C9orf72*	602	
		Total presymptomatic	775	
		Presymptomatic *GRN*	308	
		Presymptomatic *MAPT*	158	
		Presymptomatic *C9orf72*	309	
		Total symptomatic	524	
		Symptomatic *GRN*	109	
		Symptomatic *MAPT*	122	
		Symptomatic *C9orf72*	293	

Abbreviations: *GRN*, progranulin; *MAPT*, microtubule-associated protein tau; *C9orf72*, chromosome 9 open reading frame 72.
